# Pancreas Preservation: Are We Getting Warmer With Normothermic Ex Vivo Perfusion?

**DOI:** 10.1097/TP.0000000000005479

**Published:** 2025-07-22

**Authors:** Jeevan Prakash Gopal, Anand S.R. Muthusamy, Vassilios E. Papalois, Frank J.M.F. Dor

**Affiliations:** 1 Imperial College Renal and Transplant Centre, Imperial College Healthcare NHS Trust, Hammersmith Hospital, London, United Kingdom; 2 Department of Surgery and Cancer, Imperial College, London, United Kingdom; 3 Division of HPB and Transplant Surgery, Erasmus MC Transplant Institute, Erasmus MC University Medical Center, Rotterdam, the Netherlands

Pancreas transplantation remains the most effective treatment for selected patients with brittle diabetes. As the global burden of diabetes increases, so too does the demand for pancreas transplantation. In light of this, strategies to improve organ utilization and expand the donor pool—particularly through the safe use of extended criteria donors—are urgently needed. Ischemia–reperfusion injury (IRI), a major contributor to early graft loss through graft pancreatitis and thrombosis, remains a key challenge. While advanced preservation techniques—including machine perfusion in hypothermic or normothermic settings—hold clinical promise, their role in mitigating IRI and enabling viability assessment in pancreas grafts remains underexplored.

The pancreas is particularly susceptible to ischemic injury. IRI leads to microcirculatory failure and subsequent pancreatitis, with the islets of Langerhans, especially vulnerable. Several therapeutic strategies have been attempted without much success. Normothermic ex vivo perfusion (NEVP), which has shown benefit in preserving liver,^1,2^ lung,^3^ and kidney^4^ grafts, offers a potentially superior alternative to static cold storage (SCS). However, the pancreas poses unique challenges because of its low perfusion requirements and lack of end arteries. Initial efforts at machine perfusion of the pancreas resulted in edema, hemorrhage and venous congestion, largely because of high perfusion pressures and endothelial injury. Nonetheless, recent progress in perfusion technology and preservation solutions, alongside successes in other organs, has reignited interest in pancreas perfusion.

In this issue of *Transplantation*, Parmentier et al^5^ investigate the effects of NEVP in a porcine pancreas transplantation model. They compared grafts preserved with 5 h of SCS (n = 4) to those subjected to 3 h of NEVP following 2 h of SCS (n = 4). Key outcome measures included apoptosis (by terminal deoxynucloetidyl transferase deoxy uridine triphosphate nick end labeling [TUNEL] staining), oxidative stress (plasma and tissue 8-hydroxy 2-deoxyguanosine [8OHdG]), graft function (amylase, lipase, and glucose levels), and histopathologic assessment. The posttransplant follow-up lasted 3 d. Although TUNEL-positive cells were comparable at the end of preservation and 60 min postreperfusion, the NEVP group demonstrated significantly fewer TUNEL-positive cells at sacrifice (*P* < 0.001), suggesting reduced apoptosis following reperfusion. This is notable even in the setting of minimal cold and warm ischemia times, raising the possibility that NEVP may reverse early apoptotic changes—particularly relevant for extended criteria donor grafts.

Regarding oxidative stress, plasma 8OHdG levels did not differ at baseline or postoperative day 1 but were significantly lower in the NEVP group on day 3 (*P* = 0.01). Tissue staining corroborated these findings. Although the fold change in 8OHdG was not statistically significant (1.26-fold versus 1.01-fold; *P* = 0.29), the trend favors NEVP’s ability to stabilize oxidative stress, with likely greater impact in a larger sample size. The use of 8OHdG as a novel biomarker of oxidative DNA damage in the context of ex vivo pancreas perfusion and the use of a dialysis cassette to limit graft edema are noteworthy innovations.

Histologically, the NEVP group exhibited less parenchymal necrosis, hemorrhage, and fat necrosis. Islet morphology was preserved in both groups. Although early metabolic function was similar on day 3, the long-term effects of NEVP on endocrine function remain to be investigated.

Compared with previous studies,^6,7^ the authors achieved improved perfusion flows (90–130 mL/min at 20–25 mm Hg) using verapamil, epoprostenol, and aprotinin. Whether this contributed to the observed rise in amylase levels during perfusion or because of the closed system perfusion remains unclear, though no adverse impact on graft function or histology was seen—consistent with other reports.^8-10^ This finding reiterates the limited utility of conventional biochemical markers such as amylase in assessing graft viability during machine preservation.

Overall, this study adds to the growing body of evidence supporting NEVP in pancreas transplantation. Although promising, successful clinical translation will require validation in larger cohorts with extended follow-up, cost-effectiveness assessments, and protocol standardization.

In conclusion, pancreas machine perfusion is both feasible and effective in porcine models. NEVP shows potential not only in preserving graft integrity but also in mitigating IRI and enhancing viability assessment. Continued investment in translational research is vital to unlock the clinical benefits of NEVP (alone or in combination with HMP) and improve outcomes for patients with insulin-dependent diabetes.
